# Explainable Machine Learning for Human Activity Recognition Using Auxetic cTPU Knee-Worn Sensors

**DOI:** 10.3390/s26175548

**Published:** 2026-08-31

**Authors:** Abeer Elkhouly, Umar Asghar, Ganga Raj

**Affiliations:** School of Engineering, University of Wollongong in Dubai, Dubai 20183, United Arab Emirates; gangar@uow.edu.au

**Keywords:** human activity recognition (HAR), auxetic materials, intelligent sensors, wearable devices, human–machine systems, explainable AI (XAI), human gait classification

## Abstract

This paper presents a wearable soft strain sensor based on a commercially available conductive thermoplastic polyurethane (cTPU) 3D-printed as an auxetic soft metamaterial for human activity recognition. The growing demand for flexible and wearable electronics, driven by advances in artificial intelligence, highlights the importance of such sensors in healthcare, medical rehabilitation, soft robotics, and human–machine interfaces. The auxetic cTPU sensor was mechanically and electrically characterized through empirical measurements and validated against numerical simulations. A single sensor mounted on a knee brace was used to collect gait signals across four activities: running, walking, standing, and sitting. Two classification approaches were investigated. A Long Short-Term Memory (LSTM) network was trained directly on the raw time-series signal, with the best configuration achieving 96% accuracy using Relative Standard Deviation Normalization with 50 hidden units. Traditional machine learning models, namely Random Forest and XGBoost, were trained on 30 extracted time-domain and frequency-domain features per motion cycle, achieving 100% and 97.33% accuracy, respectively, under five-fold cross-validation. To enhance model transparency, explainability analysis using SHAP identified power spectral density and the first harmonic frequency as the most consistently influential features across both models, with dynamic activities driven by frequency characteristics and stationary activities distinguished by signal mean amplitude. The results demonstrate that auxetic cTPU soft strain sensors combined with machine learning and explainable artificial intelligence provide an accurate and interpretable solution for wearable human activity recognition, highlighting their potential for applications in robotics, healthcare, and human–robot interfaces.

## 1. Introduction

The growing demand for continuous health monitoring and real-time motion analysis has driven significant interest in wearable sensor technologies. The global wearable devices market is projected to reach approximately 63 billion dollars by 2025, reflecting the rapid growth of inertial measurement unit technology and widespread computing, which have made wearable sensor-based human activity recognition more accessible and practical [1]. These systems have broad applications in healthcare, rehabilitation, sports performance, and elderly care, where accurate and continuous monitoring of human movement is essential.

Human Activity Recognition (HAR) using wearable sensors has become a major area of research in recent years. The field has been transformed by wearable sensors, creating new opportunities in health monitoring and fitness training, with key challenges remaining in sensor design, placement, data quality, and user compliance [2]. Traditional rigid sensors are limited in their ability to conform to the human body during dynamic activities, motivating the development of flexible and stretchable sensing solutions that can capture complex motion patterns without restricting movement.

Among emerging sensing approaches, auxetic structures, characterized by a negative Poisson’s ratio and the ability to expand laterally under tension rather than contract, offer a geometry-driven means of enhancing strain sensitivity and conformability in wearable sensors [3,4]. When fabricated from composite thermoplastic polyurethane (cTPU), auxetic sensors combine this geometric sensitivity advantage with the flexibility, durability, and conductivity benefits of the material itself, making them well suited to capturing joint movements such as knee bending and extension.

In this study, an auxetic cTPU-based soft sensor is integrated into a stretchable knee brace to capture knee joint angle amplitude and frequency during movement. The resulting sensory data is used to train machine learning and deep learning models for the classification of everyday activities, including standing, walking, running, and sitting. To improve model transparency and clinical relevance, the study further incorporates a temporal saliency analysis of the LSTM classifier alongside explainable AI (XAI) analysis, enabling interpretable insight into the features driving activity classification. This combination of auxetic cTPU sensing, deep learning, and explainability addresses a gap in current wearable HAR research, where flexible, knee-worn sensors and interpretable classification have rarely been integrated together.

## 2. Related Work

### 2.1. IMU-Based Estimation of Joint Kinematics and Movement Classification

A substantial body of work has used Inertial Measurement Units (IMUs) with machine learning to estimate joint angles and support continuous, out-of-lab movement analysis, addressing the limitations of laboratory-based motion capture. Neural network architectures, particularly recurrent variants, have proven effective for this task: Alemayoh et al. [5] found that a Bidirectional LSTM outperformed alternative models for lower-limb angle estimation from a single IMU, while Niño-Tejada et al. [6] similarly showed a CNN-LSTM hybrid outperforming BLSTM for shoulder/elbow angle estimation, though both studies noted markedly higher error for complex rotational movements than for planar ones. Focused specifically on the knee, Yin et al. [7] used a PCA-based algorithm on gyroscopic data from flexible inertial sensors to estimate the knee flexion/extension axis, while Tham et al. [8] and Hossain et al. [9] developed dynamic and deep-learning-based networks to estimate 3D knee angle and ground reaction forces across varied gait conditions. These studies establish that IMU + ML pipelines can achieve clinically useful accuracy for knee kinematics, but they address angle estimation (regression) rather than movement classification and rely on rigid sensor hardware.

Beyond kinematic estimation, IMU data combined with machine learning has been widely applied to classify discrete movements or activity phases across clinical and sports contexts. Applications include classifying patient body position in hospital beds [10], monitoring Achilles tendon load and gait during injury recovery [11,12], and identifying movement phases in sports such as fast bowling, taekwondo, and basketball [13,14,15,16]. Reported classification accuracies are generally high (85–98%), with ensemble and boosting methods (XGBoost, Ensemble Bagged Trees) frequently outperforming simpler classifiers. Notably, two of these studies [12,16] used SHAP analysis to identify influential signal features, pointing to a broader need for interpretability in this domain. However, none of these studies address the knee specifically, rather than angle estimation or sports-phase classification, and all rely on rigid IMU hardware rather than flexible, skin-conformal sensors.

### 2.2. Flexible Sensors for Movement and Knee Monitoring

Because rigid IMUs constrain comfort and natural movement, recent work has explored stretchable and textile-based sensors as alternatives for continuous human activity monitoring. Examples include a triboelectric stretch sensor for spine motion [17], a textile capacitive strain sensor for yoga posture classification (94.4% accuracy) [18], and a ferroelectret nanogenerator-based kinesiology tape for monitoring arm strength and rehabilitation progress [19]. These studies demonstrate that flexible sensors can match rigid-sensor classification performance while improving wearability, targeting the spine, arm, and general posture rather than the knee.

A smaller set of studies has applied flexible sensing specifically to the knee. Chen et al. [20] designed a two-layer fabric strain sensor array with eight sensing elements around the knee, and found that a hybrid BiLSTM + Attention model outperformed standalone CNN and LSTM models (95% vs. lower accuracy) for activity recognition—an early indication that attention mechanisms offer a meaningful advantage for this sensing modality. Wang et al. [21] developed a 3D-printed, electrospun thermoplastic polyurethane (TPU)-based iontronic tactile sensor capable of mapping spatial pressure distribution around the knee via a 5 × 5 sensor array. Ma et al. [22] introduced SyncKnee, a multi-channel strain-gauge sensor for osteoarthritis monitoring, classifying five knee behaviors with a Random Forest model at 98.48% accuracy. Together, these studies confirm the feasibility of flexible, knee-specific sensing with strong classification performance, and identify TPU as a promising sensing material, but only one study [20] incorporates attention, and none report explainability.

Beyond material choice, sensor geometry has also been explored as a means of enhancing sensitivity. Auxetic structures, characterized by a negative Poisson ratio, have emerged as a promising approach for flexible strain sensing in wearable applications. Unlike conventional materials that contract laterally when stretched, auxetic metamaterials exhibit unconventional mechanical behavior such as lateral expansion when stretched and lateral contraction when compressed, making them highly versatile for advanced sensing applications [3]. Auxetic mechanical metamaterials offer a geometry-driven solution to the fundamental limitations of conventional positive Poisson’s ratio materials, and are particularly suitable for resistive tactile sensors used in robotics, artificial limbs, and wearable systems [4]. This unique property significantly enhances the sensitivity and conformability of wearable sensors to the human body. Wearable strain sensors based on auxetic structures play a pivotal role in real-time human motion detection and health monitoring. Graphene-modified auxetic fabric sensors exhibit an eight-fold improvement in sensitivity compared to conventional plain fabric sensors, with the auxetic structure enhancing sensitivity by synchronizing resistance changes in multiple directions [23]. Furthermore, when integrated with machine learning, auxetic strain sensors have demonstrated recognition accuracy of more than 98% for rehabilitation training, including real-time classification of different knee bending angles, highlighting their significant potential for wearable human–machine interaction [24]. The ability of auxetic structures to expand laterally under tensile loading makes them especially effective for capturing joint movements such as knee bending and extension. In our previous work [25], we developed an auxetic cTPU-based soft strain sensor and demonstrated its feasibility for AI-driven gait analytics. That study did not include a full electromechanical characterization of the sensor, nor did it explore deep learning-based classification, model explainability, or interpretability analysis. The present work substantially extends [25] by (i) providing comprehensive mechanical and electrical characterization of the sensor (stress–strain response, gauge factor, and piezoresistive behavior); (ii) introducing an LSTM-based deep learning classifier alongside traditional machine learning (Random Forest, XGBoost); and (iii) incorporating explainability at two complementary levels—SHAP analysis for feature-level interpretation and temporal saliency analysis for time-resolved interpretation of the LSTM’s decisions.

### 2.3. Explainable AI in Wearable and Biomedical Sensing

As machine learning models applied to wearable sensor data grow more complex, there is increasing emphasis on explainability—understanding which signal features drive a model’s predictions—particularly in clinical and biomedical contexts where trust, transparency, and regulatory compliance are essential. Within the sports and movement-monitoring literature reviewed above, SHAP analysis has already been used to identify influential signal features, such as tendon load indicators [12] and axis-specific motion data [16], though in both cases explainability was a secondary analysis rather than a core design objective. More recent work has begun integrating explainability directly into model architecture: Lamaakal et al. [26] developed a tiny inertial transformer for Human Activity Recognition via multimodal knowledge distillation, using attention rollout for interpretability and achieving over 98% accuracy while remaining compact and fast enough for real-time deployment on edge devices. Similarly, Navakauskas and Dumpis [27] compared LSTM, GRU, and FIRNN architectures for HAR across 16,500 trained models, finding LSTM most accurate (98.76%) but FIRNN least computationally costly, and applied Layer-wise Relevance Propagation to show that gyroscope Y-axis data was consistently the most informative feature, with interpretability patterns differing notably between architectures. Despite this growing recognition, explainability remains underexplored in flexible, skin-conformal sensor applications specifically, and no study identified in this review applies XAI methods to a TPU-based knee movement sensor.

Collectively, the existing research shows that IMU- and flexible-sensor-based systems perform well for knee kinematic estimation and movement classification [5,6,7,8,9,10,11,12,13,14,15,16,17,18,19,20,21,22], that attention mechanisms consistently improve classification accuracy over standalone CNN or LSTM models [20,26], and that explainability methods such as SHAP, attention rollout, and LRP [12,16,26,27] are gaining traction for building clinical trust. However, these advances have developed largely in isolation from one another: existing knee-specific flexible sensors [20,21,22] rarely use interpretability, and the explainability methods above have been applied mainly to general activity recognition, not to flexible, knee-specific sensors. To the best of our knowledge, the integration of XAI with an auxetic cTPU-based knee sensor for activity classification (e.g., standing, walking, running, and sitting) using knee joint angle amplitude and frequency has received limited attention in the literature. This study addresses this gap by extending our previous auxetic cTPU sensor design [25], integrated into a stretchable knee brace, combined with machine learning classification, temporal saliency analysis, and XAI analysis for accurate and interpretable activity-type classification.

## 3. Materials and Methods

**Figure 1:**
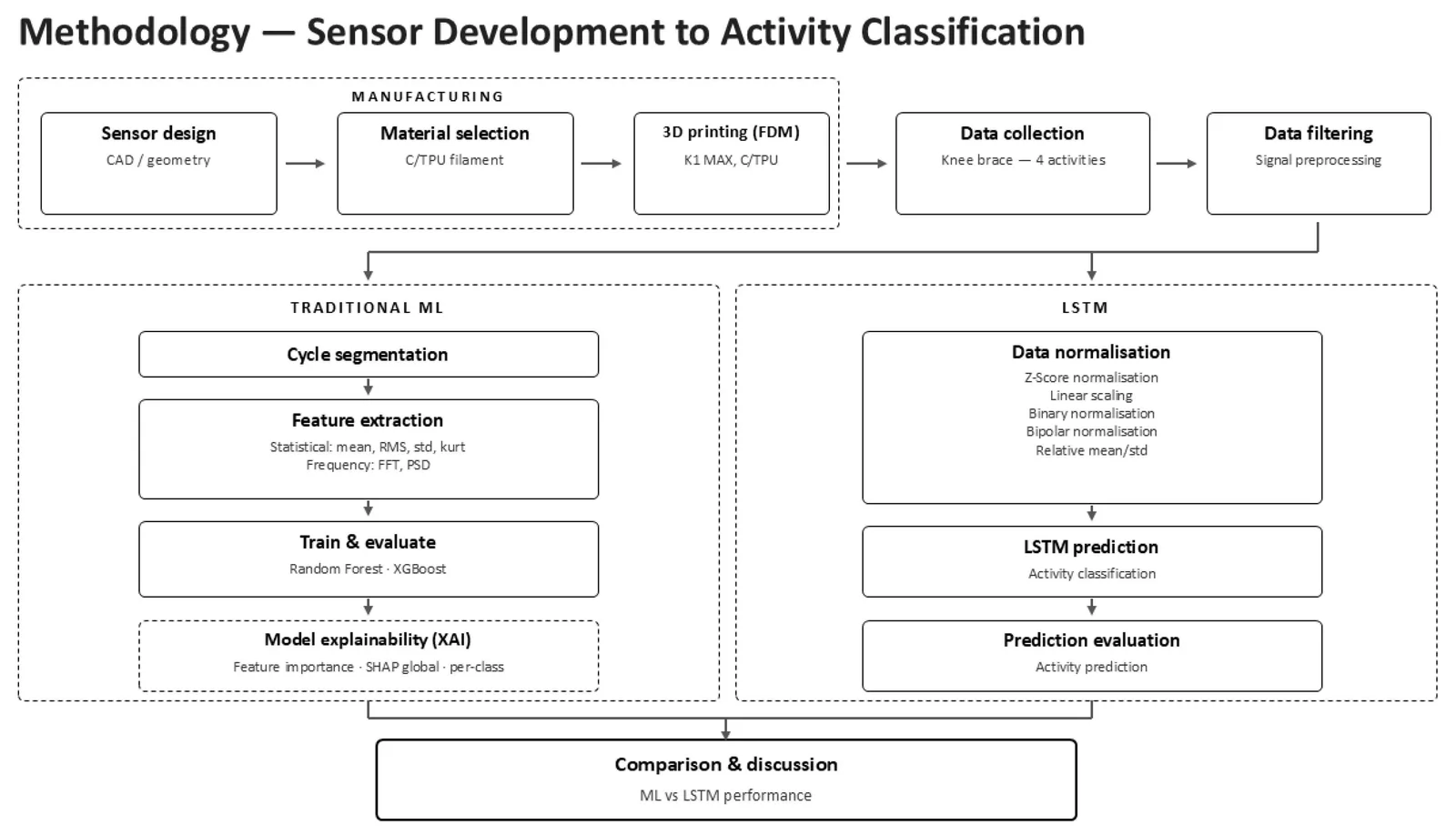
Research methodology workflow from sensor manufacturing to ML and LSTM-based activity classification and evaluation.

This section presents the methodology adopted in this study. The research follows a structured workflow, as illustrated in Figure 1, beginning with the design and fabrication of the auxetic soft sensor through material selection and 3D printing. Following fabrication, data is collected using the sensor integrated into a knee brace and filtered for signal preprocessing. The workflow then branches into two parallel approaches. In the traditional ML approach, motion cycles are segmented, features are extracted, models are trained, evaluated for performance, and interpreted using explainable AI (XAI) techniques to identify the most influential features driving classification. In the LSTM approach, data undergoes normalization before being used for activity prediction and evaluation, with a temporal saliency analysis applied to support interpretability. Finally, the results of both approaches are compared and discussed to determine the most effective method for Human Activity Recognition.

### 3.1. Sensor Design and Geometry

**Figure 2:**
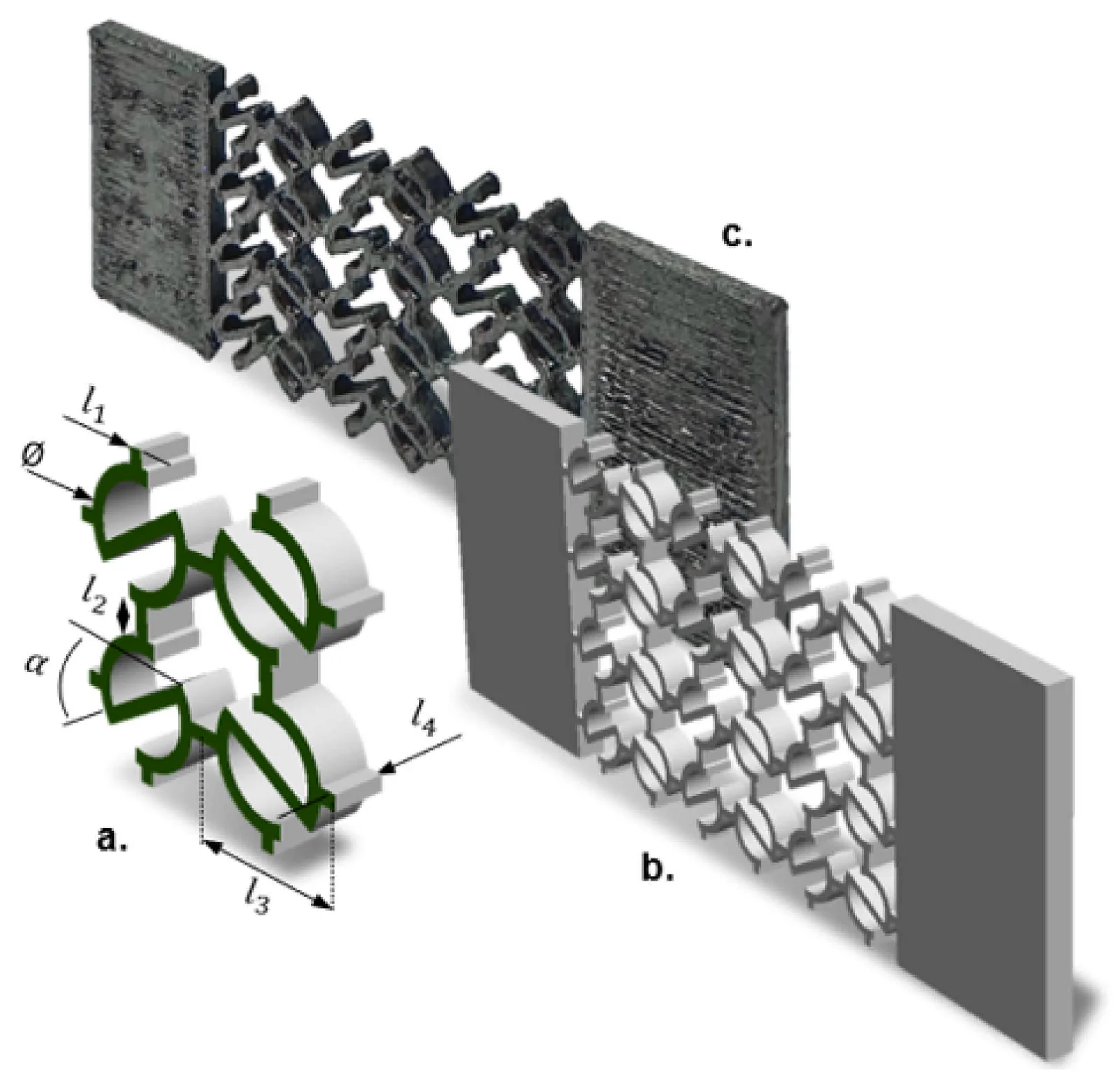
The auxetic soft sensor design: (**a**). Auxetic unit dimensions: $l_{1}=0.60\;\mathrm{mm}$, $l_{2}=1.25\;\mathrm{mm}$, $l_{3}=6.20\;\mathrm{mm}$, and $l_{4}=2.00\;\mathrm{mm}$, α=30°, and ⌀=5.00mm; (**b**). CAD model; and (**c**). Three-dimensionally printed.

The auxetic soft sensor was designed to deliver a highly flexible and strain-sensitive conductive response, as illustrated in Figure 2. The sensor geometry was developed using Fusion 360 (Autodesk) CAD software (Version 2704.1.36), incorporating an auxetic unit cell structure arranged in a 2 × 3 configuration, as shown in Figure 2b. The auxetic architecture was selected due to its negative Poisson’s ratio behavior, which allows the structure to expand laterally under tensile loading, enhancing strain sensitivity compared to conventional sensor geometries. The geometric parameters of each unit cell, shown in Figure 2a, were defined as follows: strut length $l_{1}=0.60\;\mathrm{mm}$, $l_{2}=1.25\;\mathrm{mm}$, $l_{3}=6.20\;\mathrm{mm}$, and $l_{4}=2.00\;\mathrm{mm}$, with a re-entrant angle α=30° and a node diameter ⌀=5.00mm. These parameters were carefully selected to control the mechanical deformation behavior and ensure repeatable strain response across the sensor structure. Further details on material characterisation, Finite Element Analysis (FEA), and material modeling are provided in Ref. [25]. The final 3D-printed sensor is shown in Figure 2c.

### 3.2. Material Selection and Fabrication

The sensor was fabricated using a commercially available Carbon-based Thermoplastic Polyurethane (C/TPU) elastomer, specifically Conductive Filaflex filament supplied by Recreus Industries S.L. This material was selected based on its combined mechanical and electrical properties, which are critical for soft sensing applications. The material exhibits a Shore hardness of 92A, indicating a high degree of flexibility suitable for large deformation sensing. The surface electrical resistivity ranges from 0.9 to 16.5 Ω·cm, providing adequate conductivity for resistance-based strain measurements.

The sensor was manufactured using an open-source Fused Deposition Modeling (FDM) 3D printer, specifically the K1 MAX (Creality 3D Technology Co., Guangdong, China), using the C/TPU filament. Slicing and print optimization were performed using Creality Printer software. The key printing parameters are summarized in Table 1.

**Figure 3:**
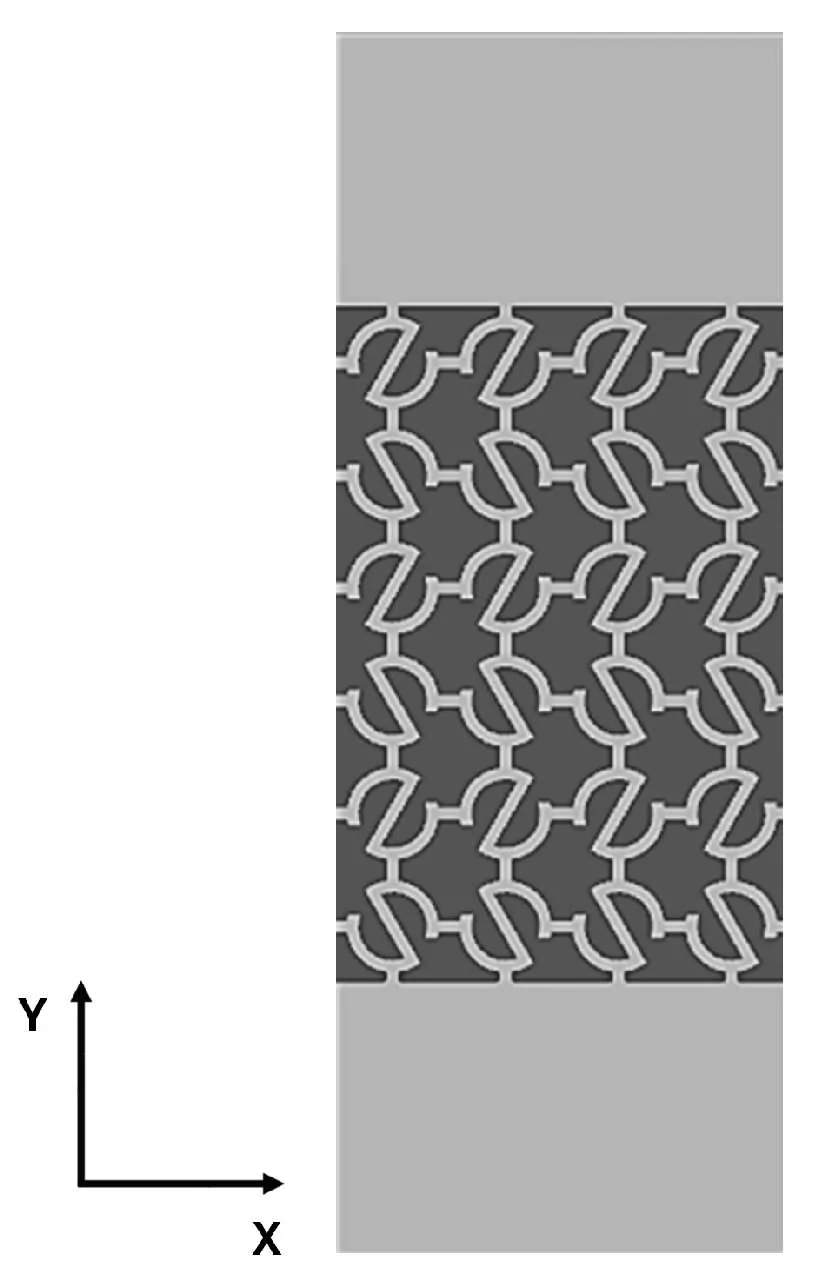
Auxetic soft sensor print orientation.

The sensor was printed flat on the build plate in the orientation illustrated in Figure 3. This orientation was deliberately chosen to ensure that the load-bearing struts are deposited in the plane of highest interlayer adhesion, which is critical for maintaining mechanical integrity under cyclic strain conditions.

### 3.3. Data Collection and Activity Classification Framework

Following the fabrication process, the auxetic soft sensor was integrated into a stretchable knee brace to monitor knee joint movements during different human activities. The sensor detects strain-induced changes in the electrical resistance of the conductive auxetic structure, generated by knee joint movement during mechanical deformation. Knee movements generate strain within the sensor structure, resulting in resistance changes that are continuously recorded and used for gait prediction and activity classification using machine learning algorithms.

**Figure 4:**
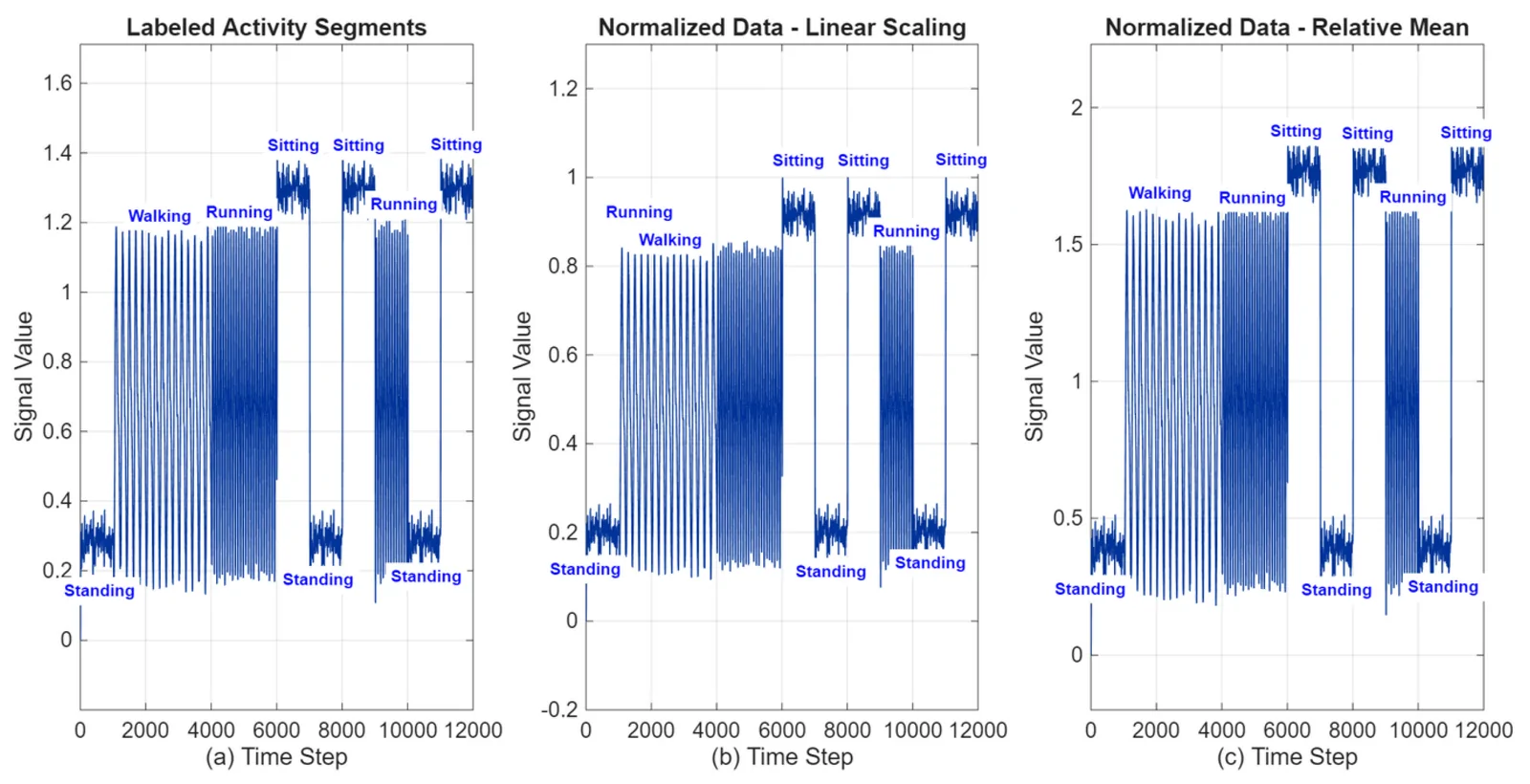
(**a**) Labeled activity segments of the raw signal. (**b**) Normalized data using linear scaling. (**c**) Normalized data using relative mean normalization.

Data were collected from a single subject performing four main activities: standing, walking, running, and sitting. The activities were recorded in the following sequence: standing, walking, running, sitting, standing, sitting, running, standing, sitting. This sequence was intentionally designed to test the model’s ability to detect transitions and classify activities accurately across different gait types. The corresponding signal waveforms are shown in Figure 4a.

The collected data was segmented into distinct activity cycles, defined by the start and end points of each repetitive movement. Each cycle was extracted and resampled to a fixed duration to facilitate feature extraction and analysis. The complete dataset consists of 12,000 data points, representing 75 distinct activity cycles across the four activity types. The 75 cycles were well balanced across the four activity classes, comprising 18 standing, 20 walking, 19 running, and 18 sitting cycles, where each cycle is represented by a single vector corresponding to the changes in electrical resistance of the auxetic soft sensor.

Following data collection, the recorded signals were pre-processed, and relevant features were extracted for machine learning analysis. The classification models were evaluated based on their ability to accurately distinguish between the four activities using the proposed wearable auxetic sensing system, as shown in Figure 1.

This study was designed as a proof-of-concept investigation, with the primary objective of carefully characterizing the sensor classifier pipeline under repeatable conditions. Data were collected from a single subject, consistent with prior studies in wearable sensor-based movement classification that have similarly relied on a limited number of subjects during initial feasibility validation [7,28], which allowed the discriminative capacity of the sensor and the extracted features to be evaluated without the confound of inter-subject variability, such as differences in gait, anatomy, or brace fit.

### 3.4. Data Pre-Processing

Data pre-processing started with noise filtering using a 4th-order Butterworth low-pass filter with a cut-off frequency of 50 Hz, applied to the sensor signal sampled at 1000 Hz. The 50 Hz cutoff was selected as a balance between removing high-frequency sensor and electrical noise and preserving the main characteristics of the signal, including transition regions between activities and the overall waveform shape needed for reliable feature extraction. The selected cutoff is also well below the Nyquist frequency (500 Hz), avoiding aliasing. Data normalization is a crucial step in pre-processing, as it improves predictive accuracy [29]. The dataset was then normalized using various techniques to standardize values and enhance model performance in human activity classification.

Z-Score Normalization transforms the data by centring it around the mean and scaling it to a standard deviation of one, ensuring a normal distribution. This technique is beneficial for human activity classification as it helps handle different activity intensities by making variations in movement comparable. Linear Scaling adjusts values within a specified range, typically between 0 and 1, while preserving relative differences [29]. It is useful for human activity classification as it ensures all sensor values are on the same scale, reducing the dominance of high-magnitude signals over lower ones.

Binary Normalization converts data into a binary format based on a threshold, making it suitable for categorical or discrete data applications. In human activity classification, this helps distinguish between two distinct movement types, such as static (sitting, standing) and dynamic (walking, running). Bipolar Normalization scales data within the range of −1 to 1, maintaining both magnitude and direction, which is particularly useful for models sensitive to polarity in values [30]. In human activity classification, this allows the model to differentiate between movements.

Additionally, Relative Mean Normalization adjusts data relative to its mean, ensuring that values are rescaled based on the dataset’s average, making it useful for datasets with different scales. This is particularly suitable for human activity classification, as it ensures variations in sensor readings are normalized, reducing reading bias. Relative Standard Deviation Normalization normalizes data by adjusting each value based on its standard deviation relative to the entire dataset, which helps in reducing variability and improving model stability [31]. In human activity classification, this technique enhances robustness by ensuring that fluctuations in movement intensity do not negatively impact classification accuracy.

The normalization techniques used in this study are defined by Equations (1)–(6), where Equation (1) corresponds to Z-Score Normalization, Equation (2) represents Linear Scaling, Equation (3) defines Binary Normalization, Equation (4) applies to Bipolar Normalization, Equation (5) corresponds to Relative Mean Normalization, and Equation (6) represents Relative Standard Deviation Normalization [32].

(1) $$x^{\prime }=\frac{x-\mu }{\sigma }$$

(2) $$x^{\prime }=\frac{\left(x-\min\right)}{\max-\min}$$

(3) $$x^{\prime }=\frac{0.8(x-\min)}{\max-\min}+0.1$$

(4) $$x^{\prime }=\frac{1.8(x-\min)}{\max-\min}-0.9$$

(5) $$x^{\prime }=\frac{x}{\mathrm{mean}}$$

(6) $$x^{\prime }=\frac{x}{\mathrm{std}}$$

Figure 4b,c illustrate the normalized data using Linear Scaling and Relative Mean Normalization, respectively, both essential for standardizing input features in human activity classification. Both methods effectively preserve the original main features of the signal, including its overall shape and transitions between activities, ensuring that classification models can accurately differentiate movement patterns while maintaining model stability and performance.

Normalization was performed on the complete dataset before partitioning the data into x1–x4 for training and testing. To verify that this approach did not introduce meaningful data leakage, the normalization parameters (e.g., mean, standard deviation, minimum, and maximum values) were subsequently recomputed separately for each partition (x1–x4) and compared against those obtained from the complete dataset. The values were found to be very close across all partitions, confirming that the influence of the held-out data on the normalization parameters was minimal and that the reported classification results are unlikely to be significantly affected.

Further data pre-processing was performed by interpolating the data to increase the number of samples. It started by estimating intermediate values between the original points. This results in a higher-resolution version of the original signal, maintaining its overall shape while improving smoothness. The function is widely used in signal processing, data resampling, and improving resolution in sampled data, making it particularly useful for applications like sensor data analysis and waveform reconstruction.

### 3.5. Classification and Explainability Framework

Each motion cycle was segmented based on activity type (standing, walking, running, or sitting) and analysed in both the time and frequency domains. Time-domain analysis: Statistical features, including average, RMS, standard deviation, kurtosis, and peak values (five minimum and five maximum peaks with locations), were extracted. These features captured signal variations and distribution. Frequency-domain analysis: The Fast Fourier Transform (FFT) was used to extract the fundamental frequency, the next two harmonics, and their locations. The normalized Power Spectral Density (PSD) was also computed to analyse frequency distribution. A total of 30 features per cycle were extracted (24 time-domain, 6 frequency-domain), forming a dataset for classification algorithms to differentiate human activities [25].

Two ensemble learning algorithms were investigated in this study: Random Forest (RF) and Extreme Gradient Boosting (XGBoost). Random Forest builds multiple decision trees independently using random subsets of data and features, with the final prediction determined by majority voting across all trees. A total of 500 trees were used to ensure stable and reliable classification. XGBoost follows a sequential boosting strategy, where each tree corrects the errors of the previous one, incorporating regularization to prevent overfitting. The model was configured with 300 boosting rounds, a learning rate of 0.05, and a maximum tree depth of 6.

Stratified five-fold cross-validation was employed to evaluate both models, ensuring every sample was tested exactly once while preserving the class distribution across folds. Performance was assessed using accuracy, precision, recall, and F1-score, alongside cross-validated confusion matrices to visualize classification behavior. Training time was also recorded to assess computational efficiency.

To improve model transparency, feature importance scores were extracted from both models, and SHAP was applied using the TreeExplainer method to explain model predictions. The analysis was carried out at two levels: globally, to show which features had the most influence across all samples, and per class, to identify the features that best distinguished each activity.

Finally, the two models were compared in terms of classification performance, training time, and interpretability, providing a well-rounded evaluation that goes beyond accuracy to support better model selection for Human Activity Recognition.

### 3.6. LSTM for Activity Classification with Temporal Saliency Analysis

This study employs a Long Short-Term Memory (LSTM) network to classify human activities based on the single-channel electrical resistance signal of the auxetic soft sensor. The dataset consists of sequential sensor readings stored in MATLAB cell arrays, where the training and test data contain time-series measurements from a single feature. Before training, the data is visualized to examine class distributions, ensuring that each activity label is well-represented.

A unidirectional LSTM network is designed for sequence classification. The model consists of a sequence input layer that takes the single-channel electrical resistance, followed by an LSTM layer with a number of hidden units to capture time-dependent patterns. To prevent overfitting, a dropout layer with a 30% dropout rate is included. The network also contains a fully connected layer with four output classes, a Softmax layer to convert outputs into probability distributions, and a classification layer to map predictions to activity labels.

The model is trained using the Adam optimizer. An initial learning rate of 0.001 is selected for stability, while a mini-batch size of 16 balances computational efficiency and performance. To mitigate overfitting, L2 regularization (0.001) is applied, and a gradient clipping threshold of 3 is set to handle exploding gradients.

Once training is complete, the model is evaluated on test sequences. Predictions are generated using the classify function, and accuracy is computed as the proportion of correctly classified labels. The accuracy of the model is calculated using the following formula:

(7) $$\mathrm{Accuracy}=\frac{\mathrm{number}\;\mathrm{of}\;\mathrm{correct}\;\mathrm{predictions}}{\mathrm{total}\;\mathrm{number}\;\mathrm{of}\;\mathrm{predictions}}\times 100$$

The predicted activities are then compared against actual recorded values, and the results are visualized in a time-step-based plot. This methodology ensures reliable activity recognition by utilizing the sequential processing capabilities of LSTM while optimizing hyperparameters for improved performance on the single-channel electrical resistance signal data.

Despite achieving competitive classification performance, LSTM networks inherently operate as black-box models, offering no explanation of which temporal regions drive their predictions. To address this limitation, a temporal saliency analysis was applied to the trained LSTM network. This analysis assigns an importance score to each time step of the LSTM hidden state, reflecting its relative contribution to the classification decision. The scores were extracted by computing the mean absolute activation across the hidden units at each time step, followed by softmax normalization to produce a normalized importance score for each time step of the Auxetic sensor signal. This approach provides post hoc interpretability analogous to attention weighting, without modifying the trained network.

## 4. Results

The results of this research are presented and discussed in the following subsections.

### 4.1. Mechanical Behavior

**Figure 5:**
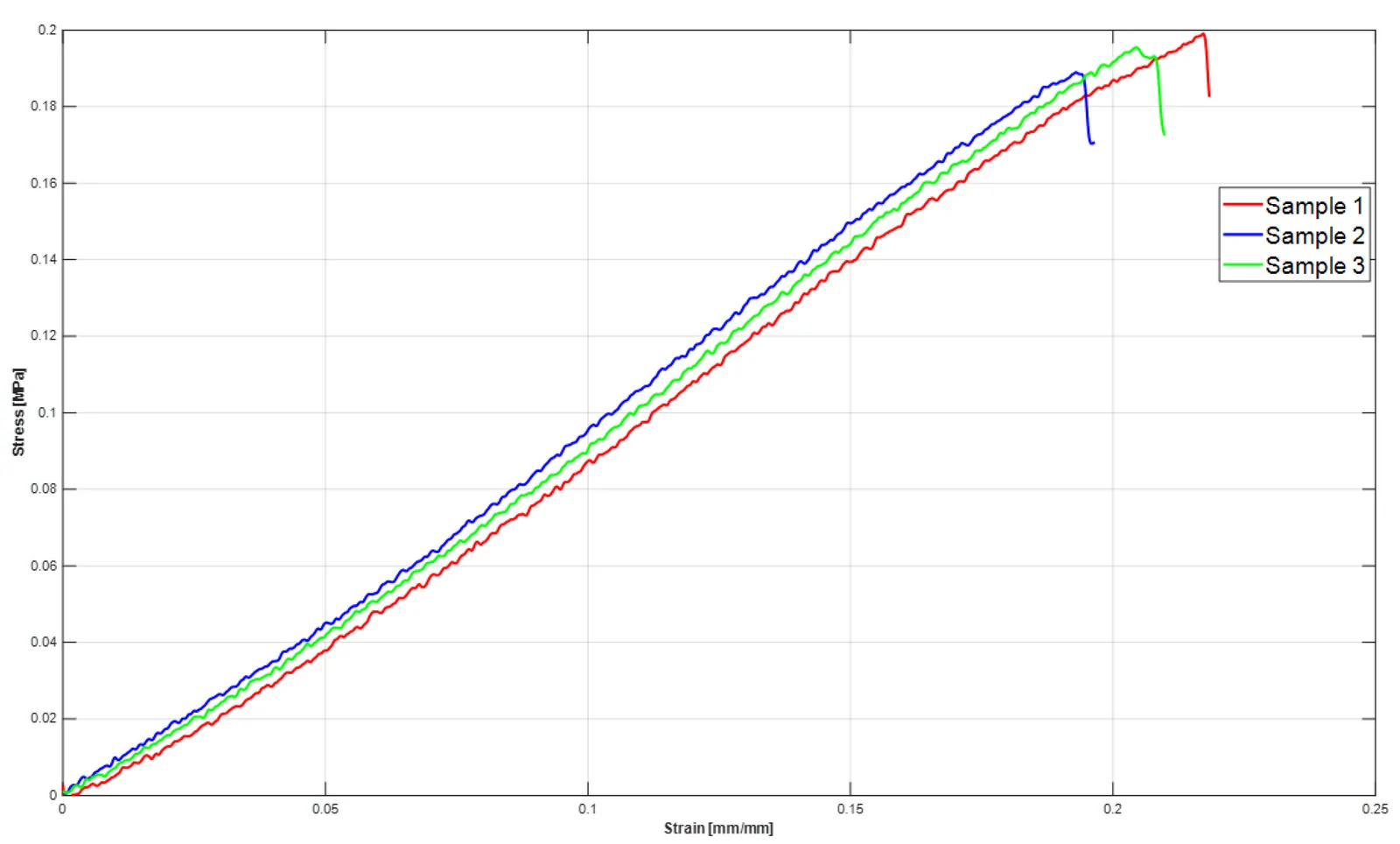
Stress–strain behavior of the auxetic sensor.

The mechanical response of the 3D-printed auxetic structure is shown in Figure 5. Here, it can be seen that the sensor demonstrates a linear elastic behavior up to a strain of approximately 0.21 mm/mm. The calculated Young’s modulus is approximately 0.95 MPa. The gradual buckling of the auxetic structure within the unit cells allows deformation with continuing increasing stress, which is consistent with negative Poisson’s ratio structures.

### 4.2. Sensor Electrical Response

**Figure 6:**
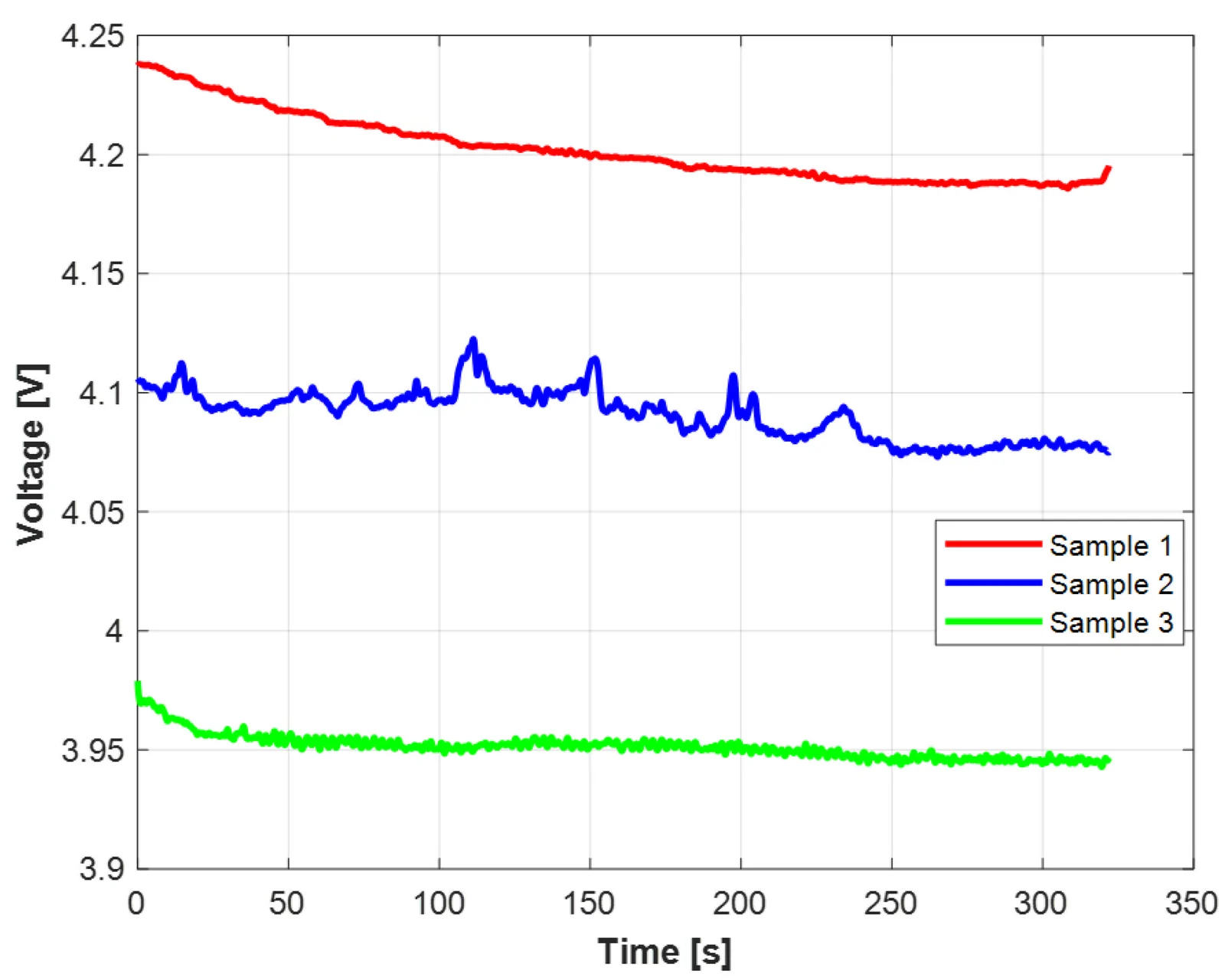
Voltage response of the auxetic sensor during straining.

**Figure 7:**
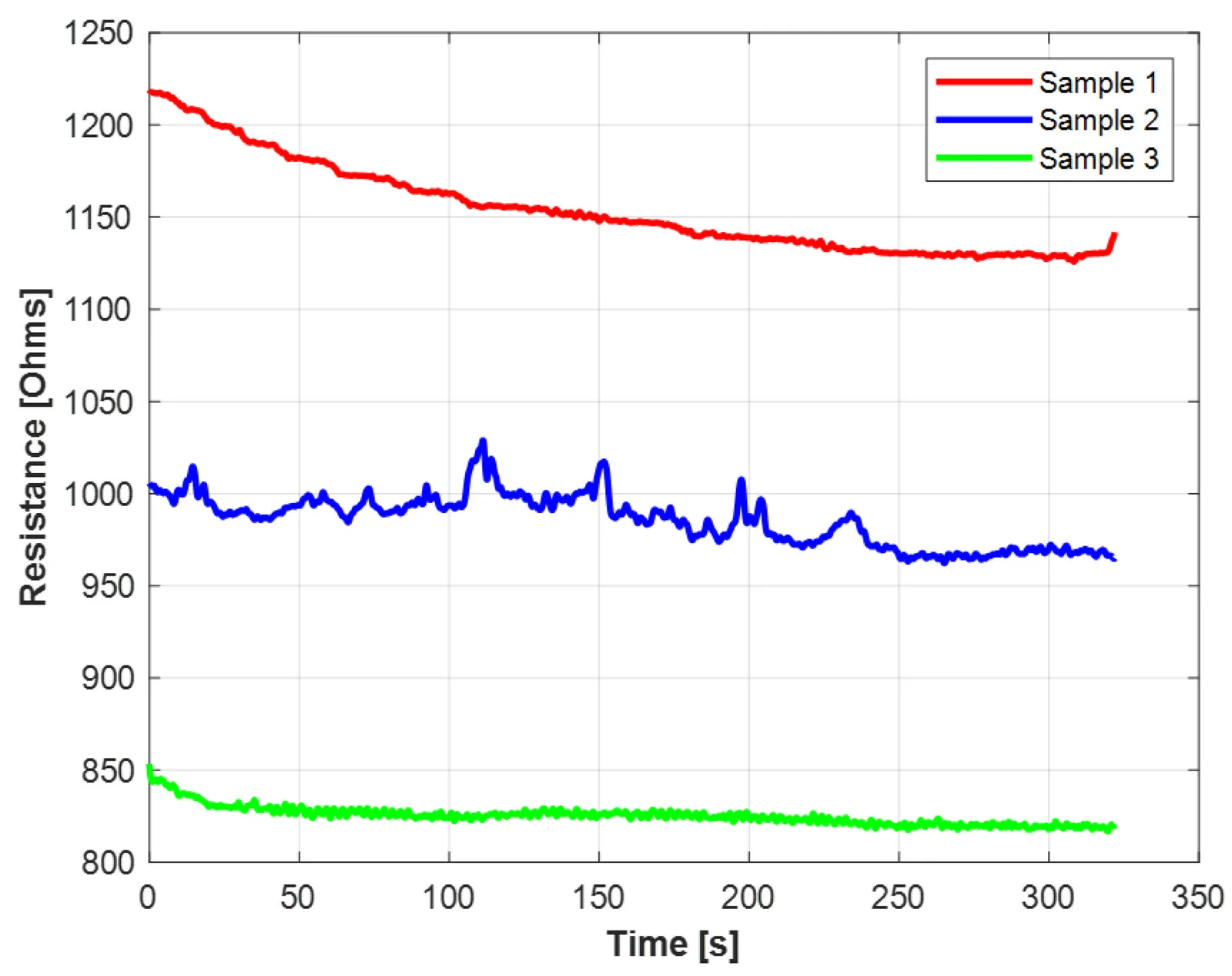
Resistance response of the auxetic sensors during straining.

Figure 6 shows the voltage output from the sensors whilst the sensors are strained. It can be seen that the voltage decreases with increasing strain. The resistance output of the sensor is shown in Figure 7. In all cases, initially, the resistance drops rapidly with the increase in tensile strain. The resistance then stabilizes, and this is when the maximum tensile strain is reached. This illustrates that once the sensor is held at a fixed stretch, the resistance stops changing, meaning the electrical connections inside the material have found a stable balance. The resistance decreases with increasing strain. Table 2 demonstrates the data collected for each sensor. A decrease in voltage correlates with the decrease in resistance, which is expected. The output voltage was acquired across the sensor. As the auxetic structure was strained, the sensor resistance decreased, resulting in a proportional decrease in the measured voltage. This relationship follows the voltage divider rule, where $V_{\mathrm{out}}=V_{\mathrm{in}}\times \frac{R_{\mathrm{sensor}}}{\left(R_{\mathrm{fixed}}+R_{\mathrm{sensor}}\right)}$, confirming that the observed electrical response is a direct consequence of the changing bulk resistance of the printed structure.

### 4.3. Piezo-Resistivity and Gauge Factor

The auxetic structure possesses a negative piezoresistive response; resistance decreases under tensile strain. This is expressed by the Gauge Factor (GF), Equation (8):

(8) $$\mathrm{GF}=\frac{\frac{\Delta R}{R_{0}}}{\varepsilon }$$

Presented in Table 2 are the auxetic sensors characterized under identical conditions. All three specimens exhibited a negative gauge factor, demonstrating a consistent direction of piezoresistive response, whereby the electrical resistance decreased with increasing tensile strain. The negative GF is expected since auxetic structures exhibit a negative Poisson’s ratio. During tensile strain, the structure stretches in both directions—longitudinal and lateral. This therefore increases the effective cross-sectional area and reduces resistance.

The coefficient of variation (CV) of the Gauge factor magnitude was approximately 38.0%, indicating that sensor-to-sensor variability remains present in the current fabrication process.

The observed variability may be associated with local conductive pathways, and contact between adjacent extruded tracks can influence the resistance–strain relationship. Nevertheless, the consistent negative piezoresistive response observed across the three independently fabricated specimens confirms that the proposed auxetic sensor architecture provides a repeatable qualitative sensing mechanism.

The present study therefore demonstrates consistency in the direction of the electromechanical response, while also identifying sensor-to-sensor sensitivity variation as an area requiring further manufacturing optimization.

### 4.4. Correlation Between Strain and Resistance

**Figure 8:**
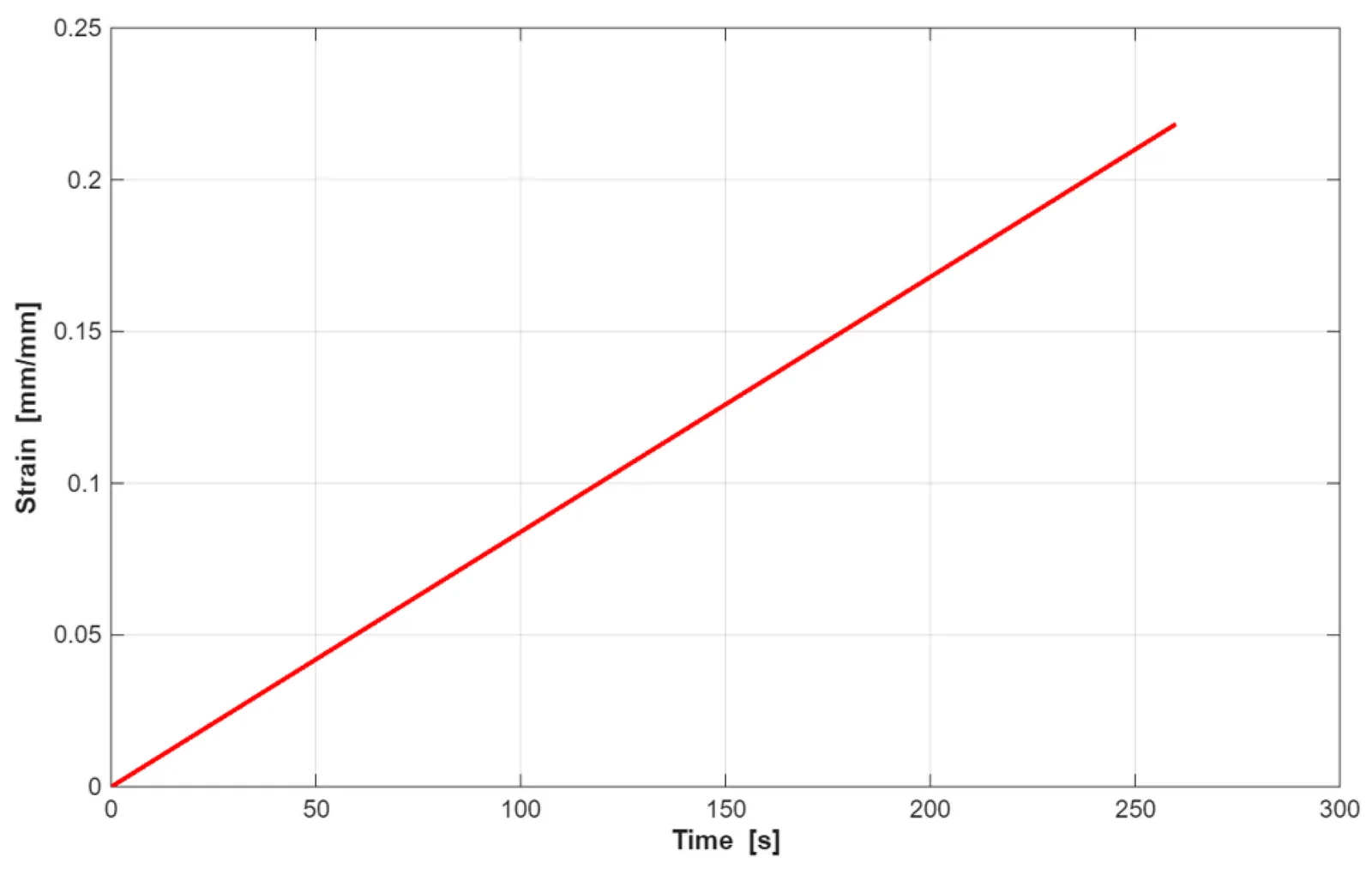
Tensile strain–time data for the auxetic structure.

**Figure 9:**
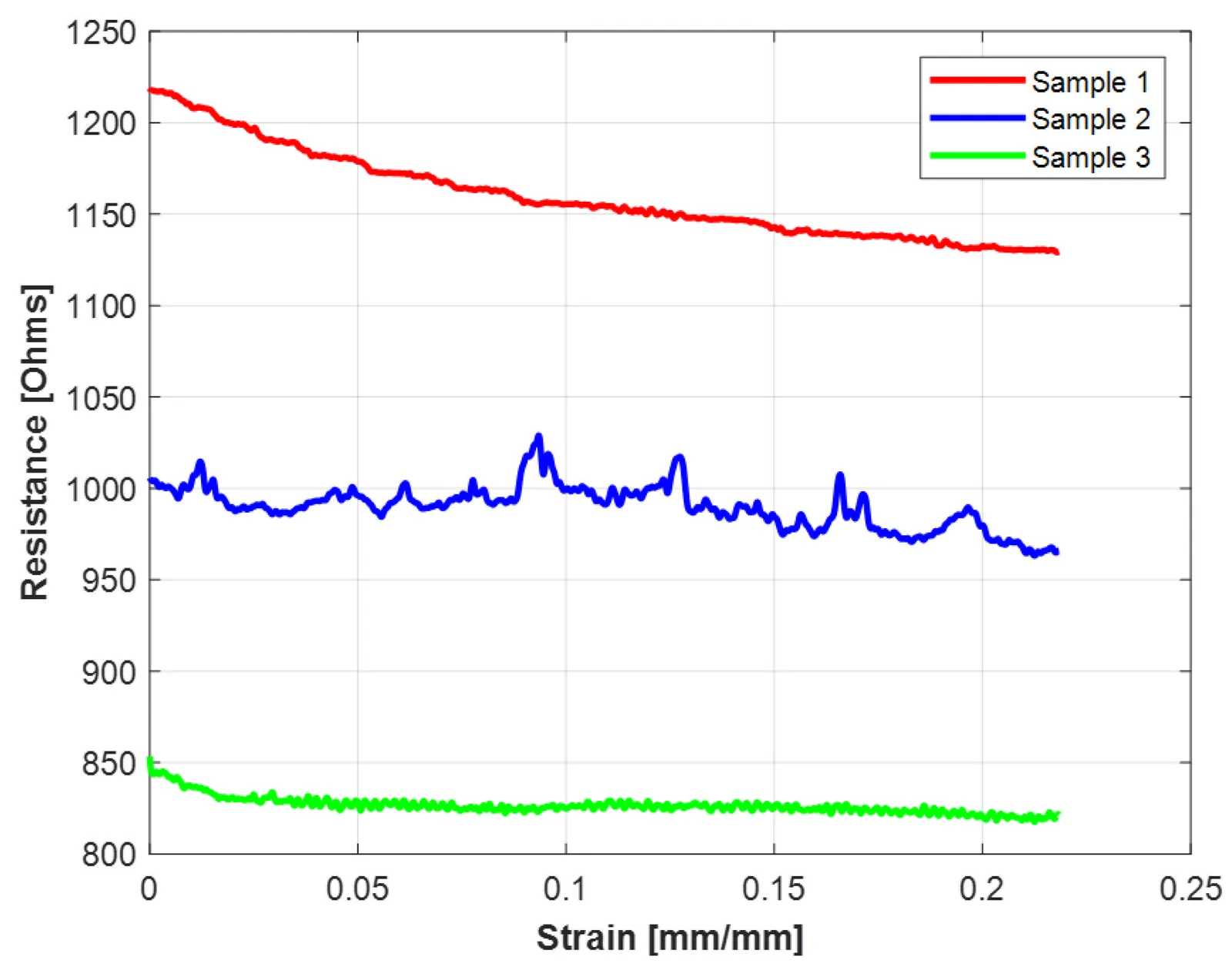
Tensile strain–resistance correlation for the auxetic structure.

By synchronizing the strain–time data (Figure 8) with the resistance–time data (Figure 9), we can directly map resistance as a function of applied strain. Figure 9 illustrates this relationship. The resistance decreases almost linearly with strain up to approximately 0.21 mm/mm, where Table 2 demonstrates the sensitivity of each sensor, which is determined using Equation (9):

(9) $$S=\frac{\Delta R}{\Delta \varepsilon }$$

Beyond this strain, the resistance plateaus, which coincides with the mechanical yielding observed in the stress–strain curve. This correlation confirms that the electrical response is mechanically driven and provides consistent piezoresistive behavior with measurable fabrication-induced variation.

The combination of auxetic mechanical behavior with negative piezo-resistivity offers unique advantages for resilient healthcare applications. A sensor whose resistance decreases under strain can be configured as a normally open fail-safe device.

Overall, these results validate the potential of auxetic 3D-printed sensors as a scalable, low-cost solution for healthcare monitoring, aligning with the objectives of resilient medical manufacturing.

### 4.5. Results of Traditional ML Methods

The classification results were obtained by training Random Forest and XGBoost on the 30 extracted features per motion cycle, covering both time-domain and frequency-domain characteristics. Both models were evaluated using stratified five-fold cross-validation to ensure reliable and unbiased performance assessment. Random Forest achieved a mean accuracy of 100% with zero standard deviation across all folds, demonstrating consistent and stable classification across all four activity classes. XGBoost achieved a mean accuracy of 97.33%, with a standard deviation of 3.27%, reflecting strong but slightly more variable performance. The performance for both models is summarized in Table 3.

**Figure 10:**
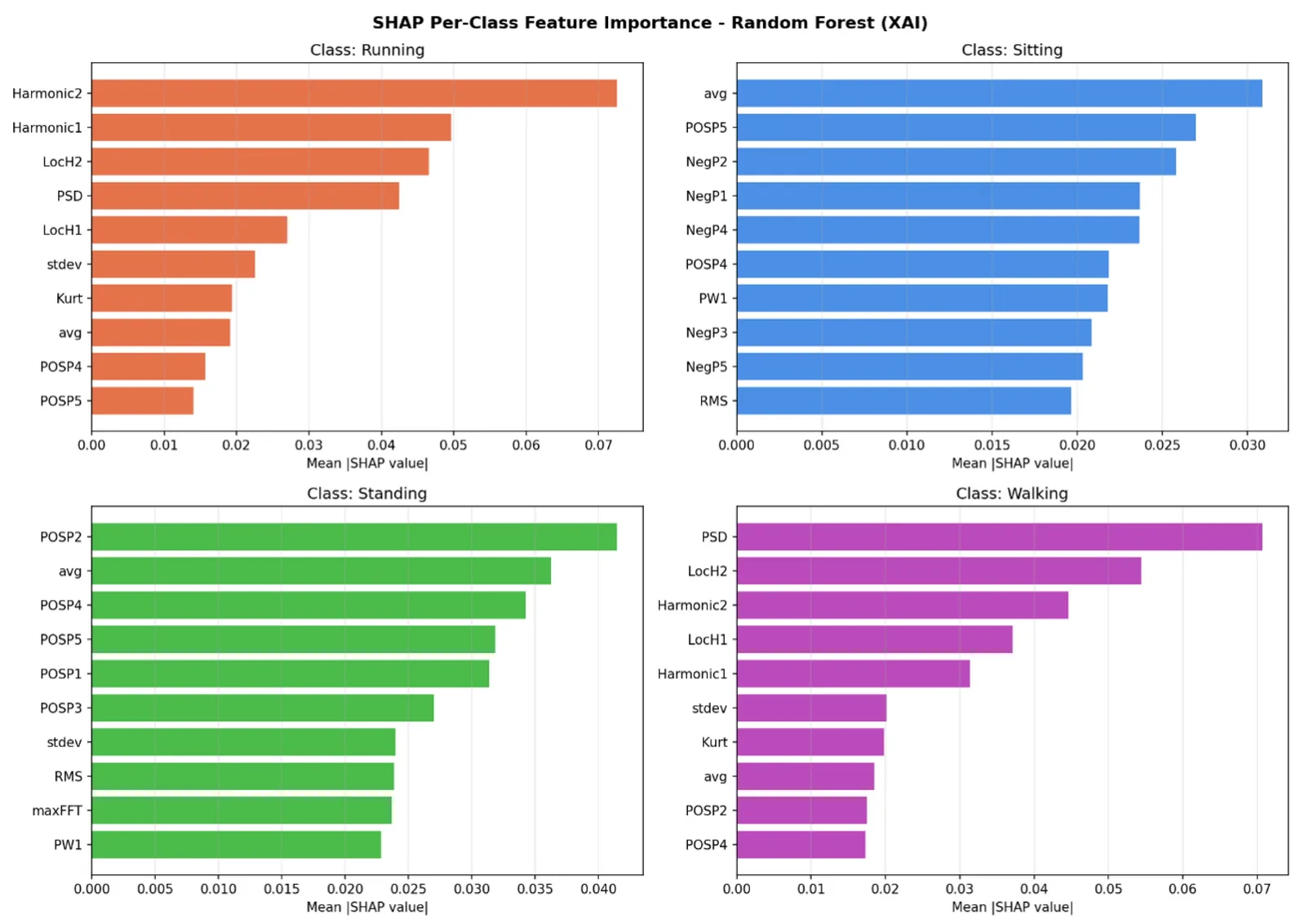
SHAP per-class feature importance for Random Forest, showing multiple contributing features per activity class (running, sitting, standing, walking).

The perfect cross-validation accuracy of Random Forest reflects the highly discriminative nature of the extracted features under controlled recording conditions. This result should be interpreted with appropriate caution, given the scale of the dataset (single subject, 75 activity cycles). The four activities occupy clearly separable regions in the feature space: dynamic activities (walking, running) are distinguished by their frequency-domain signatures, while stationary activities (standing, sitting) are separated by signal mean amplitude—a separability confirmed by the SHAP analysis (Figure 10). In addition, segmenting the 75 cycles at well-defined boundaries and resampling them to a fixed duration produced highly consistent features within each class, enabling error-free partitioning by the ensemble. The four activities were also deliberately selected to represent highly distinct movement classes, which supports class separability as the primary driver of this result rather than overfitting. The zero standard deviation across the five folds further indicates that this separation was consistently reproduced across the independent cross-validation partitions rather than being associated with a single favorable split.

**Figure 11:**
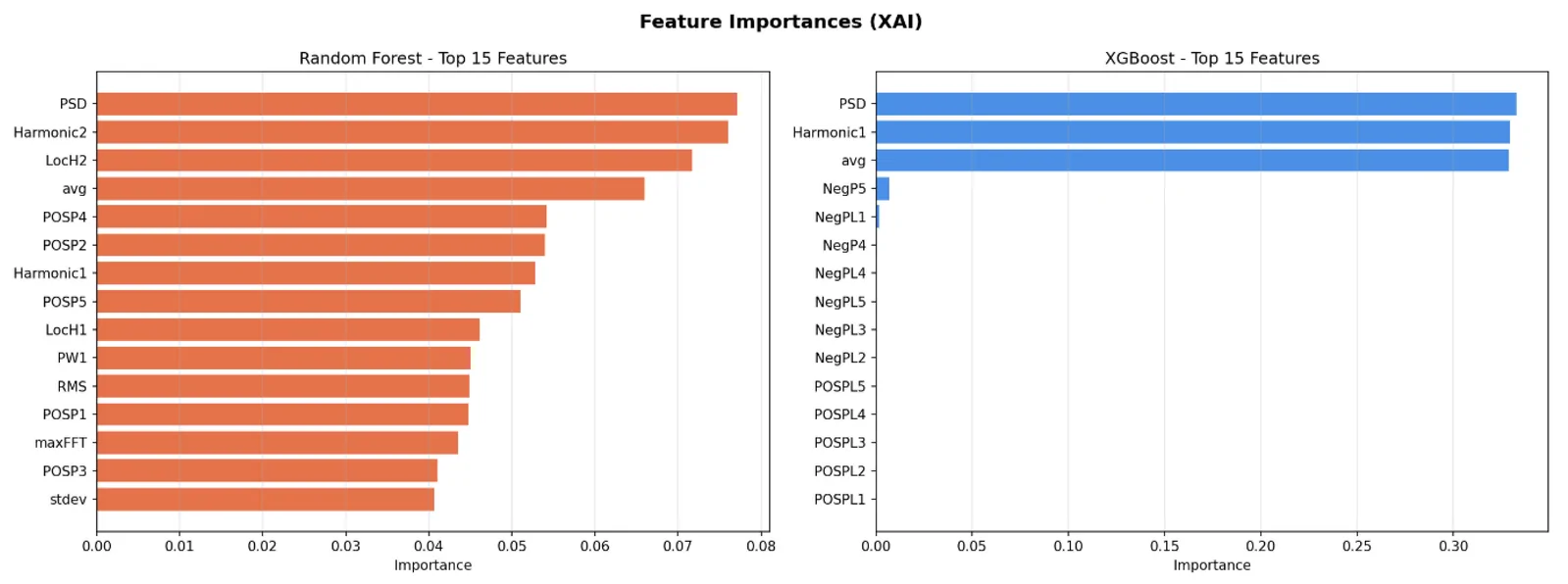
Top 15 feature importances from XAI analysis for Random Forest (**left**) and XGBoost (**right**).

Figure 11 presents the feature importance results, revealing notable differences between the two models. Random Forest spread its decisions across a wide range of features, with the power spectral density, second harmonic frequency, and its location ranked highest, showing that the model relies on a combination of both signal frequency and timing characteristics to classify activities. In contrast, XGBoost focused almost entirely on three features: the power spectral density, the first harmonic frequency, and the signal mean amplitude, with all other features contributing very little. Despite this difference, both models agreed that the power spectral density was the single most important feature, confirming that the frequency content of the signal plays a central role in distinguishing between human activities.

**Figure 12:**
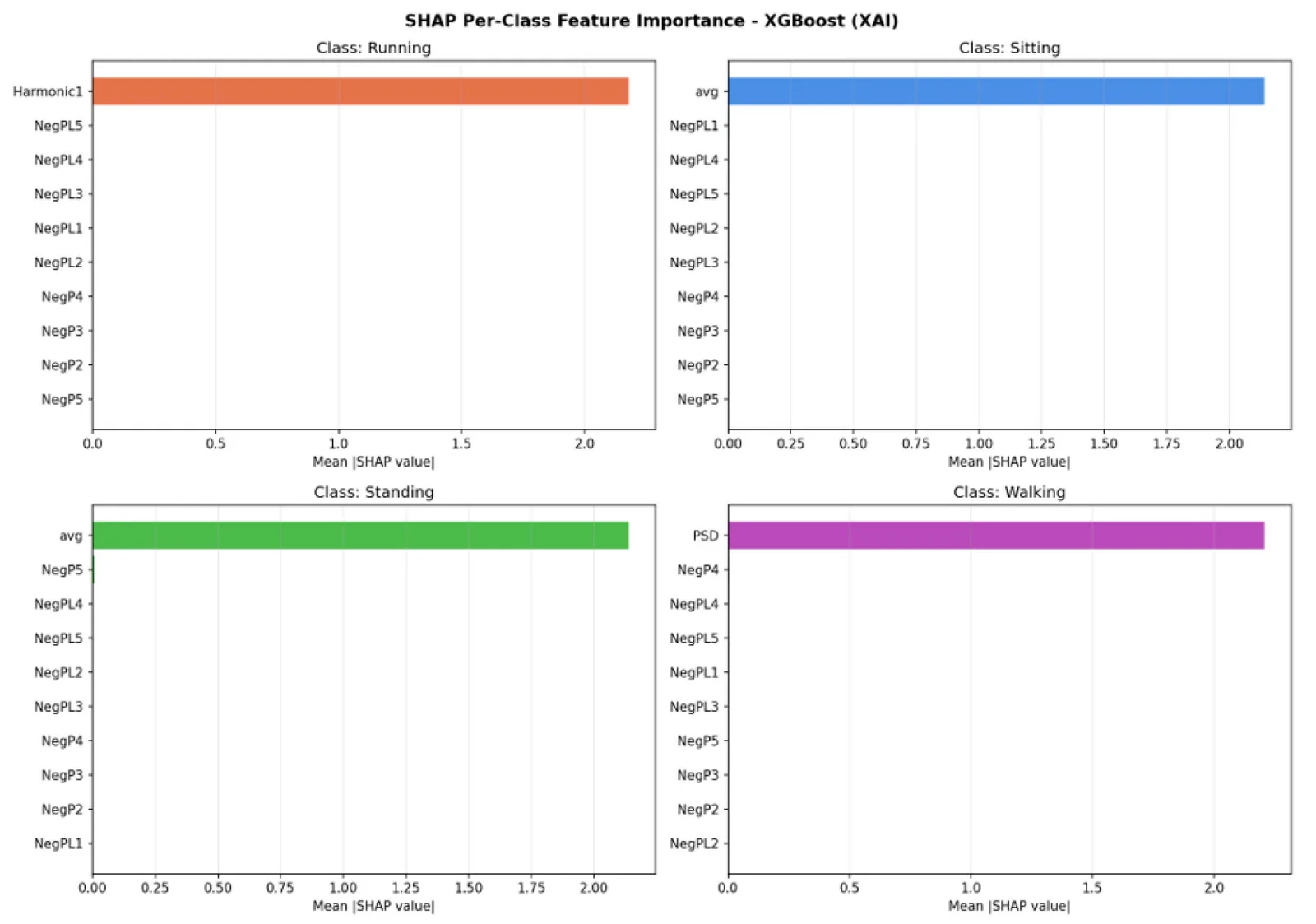
SHAP per-class feature importance for XGBoost: Harmonic1 (running), signal mean amplitude (sitting, standing), and PSD (walking) dominate each class.

The per-class SHAP analysis in Figure 12 revealed clear differences in how each model explains activity classification. For XGBoost, each activity was driven by a single dominant feature, with all other features contributing almost nothing. Running was identified primarily through the first harmonic frequency, reflecting the strong rhythmic and periodic nature of running motion. Sitting and standing were both distinguished by the signal mean amplitude, which captures the low and stable nature of stationary activities. Walking was identified through the power spectral density, suggesting that the overall frequency energy distribution is the key characteristic separating walking from other activities. This highly concentrated behavior confirms that XGBoost makes sharp, focused decisions based on one defining signal characteristic per activity.

Random Forest showed a richer and more distributed explanation across all four classes, as shown in Figure 10. For running, the second and first harmonic frequencies along with their locations were the most influential features, capturing the strong cyclic pattern of running. For sitting, the signal mean amplitude dominated alongside peak amplitude values and pulse width features, reflecting the low-energy and stationary nature of the activity. Standing was explained primarily by positive peak locations and the signal mean, indicating that the shape and position of signal peaks help distinguish standing from other low-motion activities. Walking was best explained by the power spectral density, harmonic locations, and standard deviation, combining both frequency content and signal variability to capture the moderate and rhythmic nature of walking. Overall, Random Forest provided a more comprehensive and physically meaningful explanation by distributing importance across multiple complementary features for each activity.

The training time comparison between both models: XGBoost completed training significantly faster at 0.27 s compared to Random Forest, which required 2.74 s. This highlights a practical advantage of XGBoost in time-sensitive or resource-constrained deployment scenarios, despite its marginally lower cross-validation accuracy.

### 4.6. LSTM-Based Activity Recognition Results

In this section, we explore the results of the LSTM model in the second path of the research. The dataset (x) was divided into four parts (x1, x2, x3, x4), where each part contains all types of activities but in different sequences, maintaining the overall distribution of the dataset. Three parts (x1–x3) were used for training, while the fourth part (x4) was held out for testing. The model was evaluated using short memory, medium memory, and long memory configurations, corresponding to 10, 50, and 100 hidden units, respectively.

Results are shown in Table 4 for different data analysis approaches. The results indicate the effect of normalization, memory length, and interpolation on prediction accuracy in an LSTM-based system. Memory length was tested; with 10 hidden points, accuracy ranges from 73.4% to 91.4%, with Relative Mean Normalization performing best at 91.4%. For 50 hidden points, a significant improvement was observed, with accuracy reaching 96% when using Relative Standard Deviation Normalization. Most results are above 90%, indicating that 50 hidden points provide the best balance of complexity and performance. With 100 hidden points, accuracy becomes inconsistent. While some normalizations still perform well, such as Relative Standard Deviation at 92.5%, other values drop, such as Linear Normalization at 74.8%. This suggests that increasing the memory length beyond 50 hidden points does not necessarily improve performance and may lead to overfitting or inefficiencies.

For different normalization techniques across different memory sizes, Relative Mean and Relative Standard Deviation Normalization consistently improve accuracy, particularly at 50 hidden points, where they reach 95% and 96%, respectively. Bipolar and Z-score Normalization also perform well, especially at 50 hidden points, with 95.5% and 94.4% accuracy, respectively. Binary Normalization consistently underperforms, with lower accuracy across all memory lengths. No Normalization results in lower accuracy, except at 50 hidden points (94%), where the model appears to generalize better.

Adding interpolation consistently led to a decline in model performance across all tested configurations. At 10 hidden points, accuracy decreased from 76.5% to 73%, while at 50 hidden points, accuracy dropped from 94% to 73% when interpolation was applied. The most significant decline was observed at 100 hidden points, where accuracy fell to 67%. These results suggest that interpolation disrupts the time dependencies within the data, negatively affecting the ability of the model to generalize across different activity types.

In an LSTM network, hidden units represent memory cells that store and process sequential information over time. They help capture data patterns and influence the model’s learning capacity. More hidden units may improve the ability to learn complex patterns but increase computational cost, while fewer units may lead to underfitting. The final hidden state is used for making predictions in tasks like classification and regression; it should be selected carefully, as long-term data with sudden sharp transitions will make model predictions low, such as in our model here.

**Figure 13:**
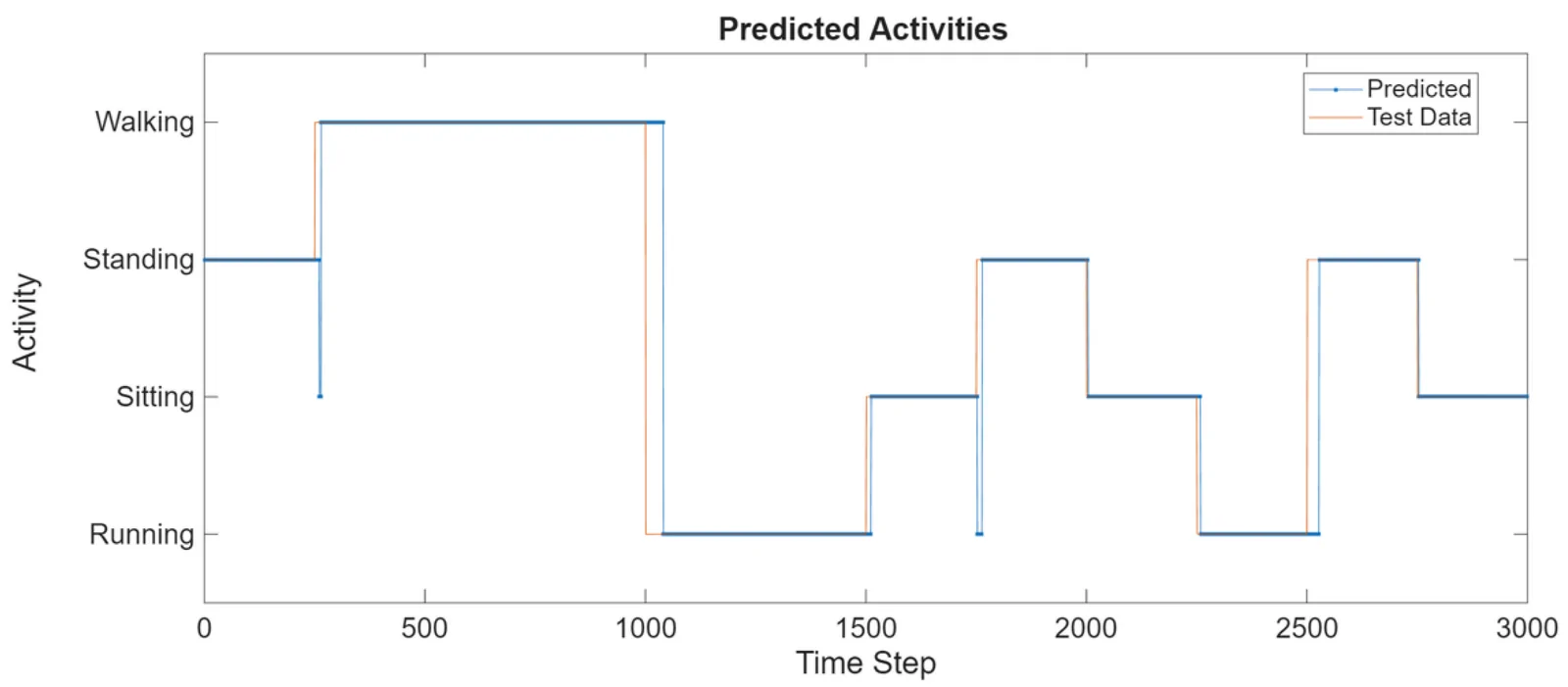
LSTM model performance on test data without interpolation, using Relative Standard Deviation Normalization and 50 hidden units.

**Figure 14:**
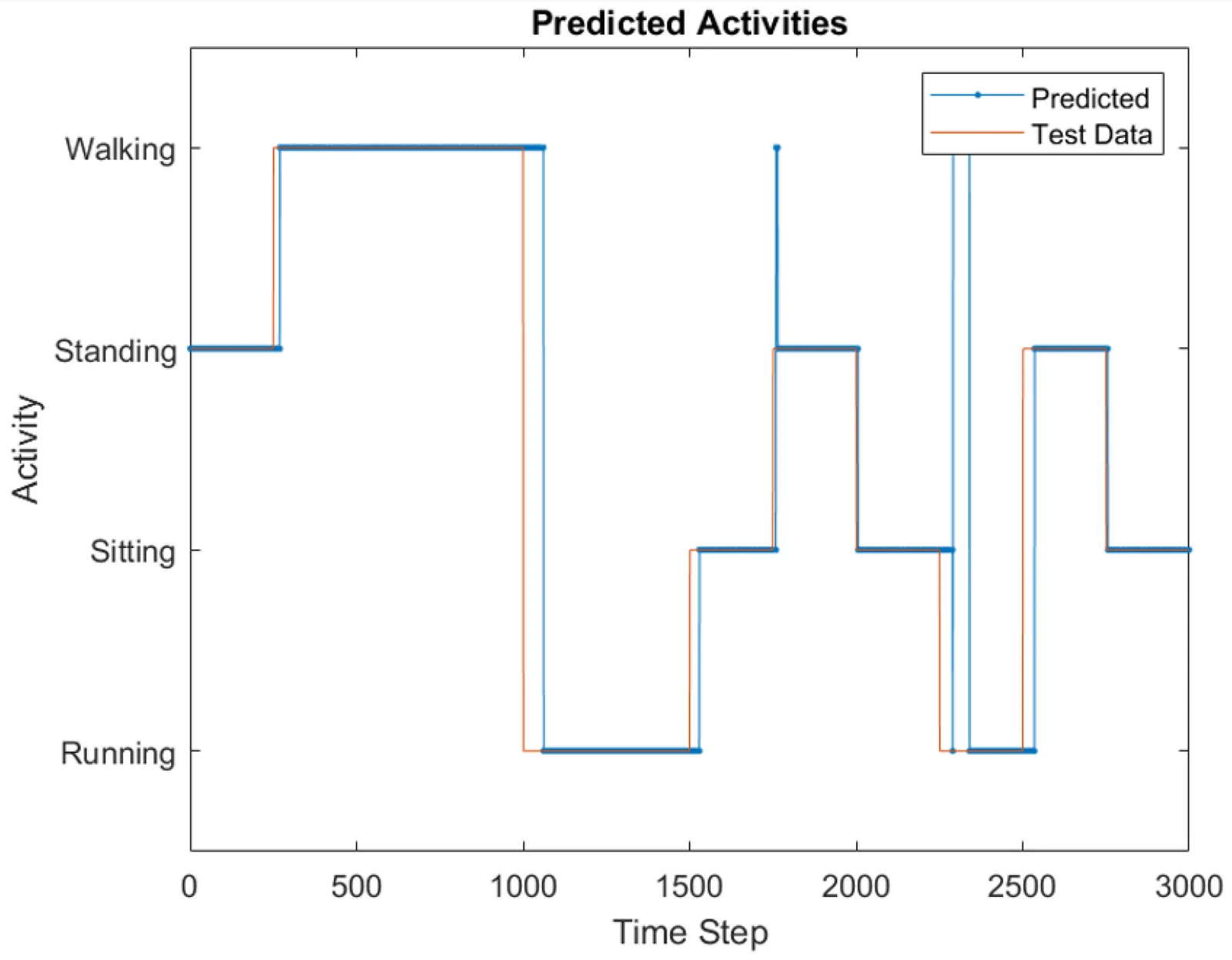
LSTM model performance on test data without interpolation, using relative mean normalization and 10 hidden units.

**Figure 15:**
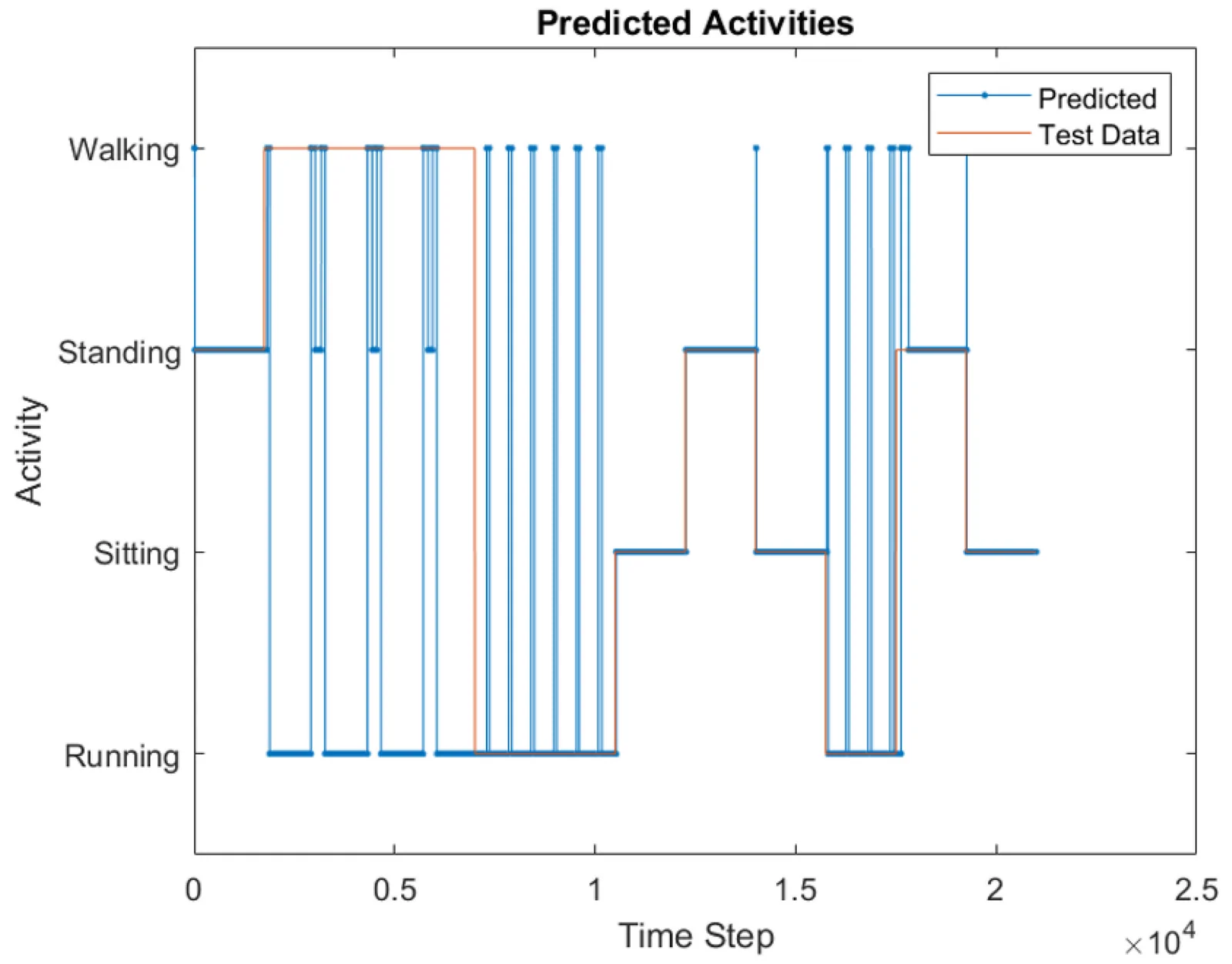
LSTM model performance on test data with interpolation, no normalization, and 50 hidden units, indicating a significant drop in performance.

An example of LSTM performance for test data: Figure 13 shows that the model with no Interpolation, Relative Standard Deviation Normalization, and 50 hidden units achieved 96% accuracy. Figure 14, the model with No Interpolation, Relative Mean Normalization, and 10 hidden points achieved 91.4% accuracy. The predictions closely align with the test data, but minor delays in transitions suggest potential areas. The model with Interpolation, No Normalization, and 50 hidden points performed significantly worse, achieving 73% accuracy, as shown in Figure 15.

To summarize, normalization significantly improved the model’s ability to detect transitions and key features of each movement, making prediction accuracy highly dependent on data normalization. The best-performing model used 50 hidden points with Relative Standard Deviation Normalization, achieving 96% accuracy. Increasing memory length to 100 hidden points did not enhance performance and, in some cases, led to overfitting or instability. Relative Mean and Relative Standard Deviation Normalization yielded the best results, whereas Binary and No Normalization produced weaker performance.

Interpolated models consistently underperformed. Interpolation was applied to assess the effect of sample size on fast transition signals and was tested for both short and long memory configurations, yielding an accuracy of 73% in both cases. However, very long memory was not suitable due to the rapid occurrence of state transitions. When tested with 200 hidden units, all models experienced a drop in accuracy to between 60% and 70%. Retaining long-term dependencies led to incorrect activity predictions, as the model struggled with rapid transitions between movements. Therefore, reduced memory length proved to be the most effective, as demonstrated in Table 3.

**Figure 16:**
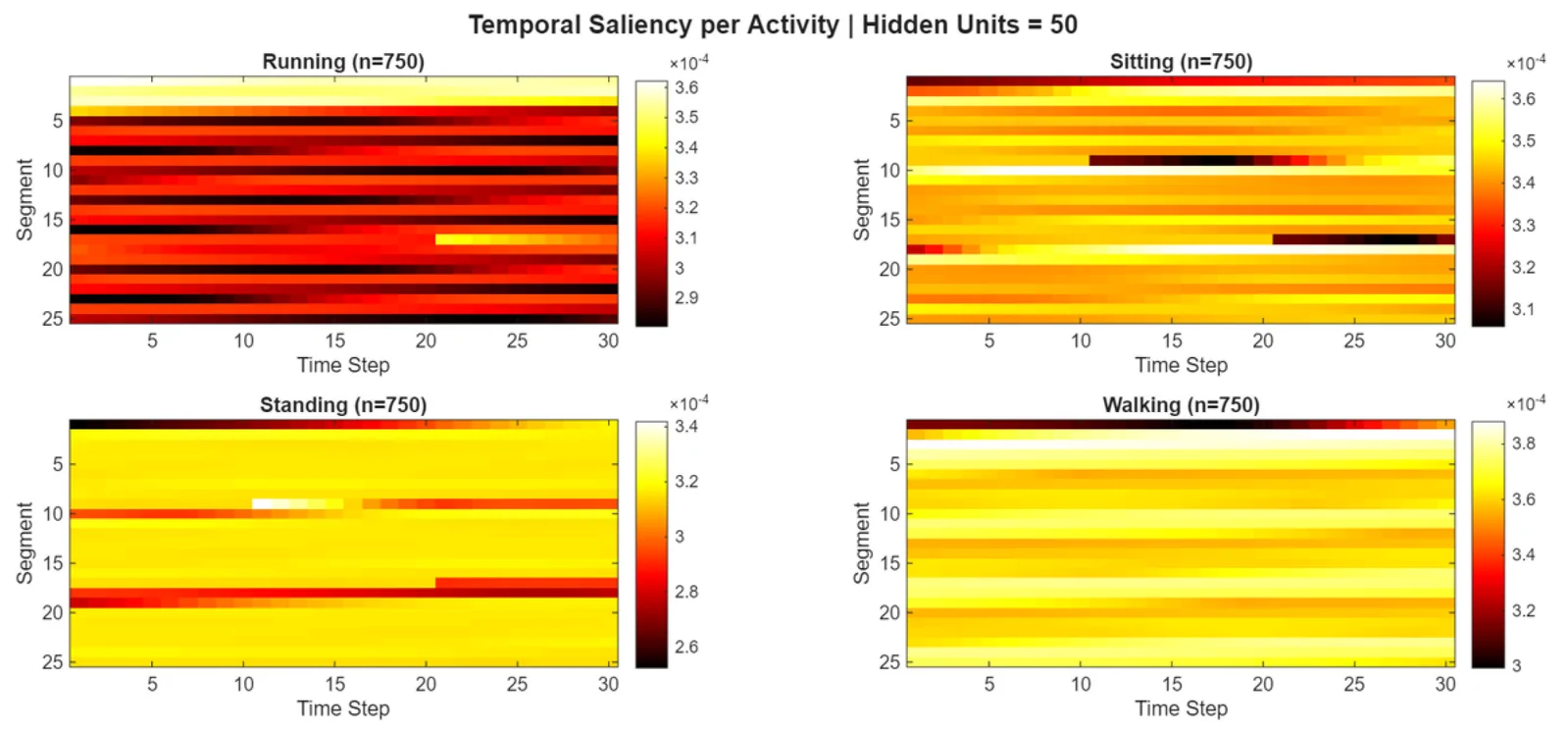
Temporal saliency heatmaps per activity (LSTM, 50 hidden units). Dynamic activities show alternating saliency bands; stationary activities show largely uniform saliency.

The temporal saliency scores extracted from the trained LSTM network show different patterns for each activity, as illustrated in Figure 16. For running, the model focuses on alternating regions of the signal, which matches the repetitive nature of running where the body moves in a consistent left-right stride pattern. For walking, a similar but more regular alternating pattern is observed, as the model captures the consistent step-by-step movement. For sitting, the saliency is mostly uniform across the signal, which is expected since sitting involves very little movement, and the model ignores most time steps as they carry little useful information. For standing, the saliency is also largely uniform but with a few specific moments of high focus, likely corresponding to small body movements or weight shifts that help the model distinguish standing from other activities. Overall, the saliency analysis shows that the model correctly identifies the most relevant moments in the sensor signal for each activity, confirming that the classification decisions are physically meaningful and not random.

## 5. Conclusions

For traditional machine learning, Random Forest and XGBoost were trained on 30 time-domain and frequency-domain features, achieving 100% and 97.33% accuracy, respectively, under five-fold cross-validation. Combining time-domain features for fast processing with frequency-domain features for improved accuracy proved particularly effective, suggesting potential for high-precision applications such as medical monitoring systems. SHAP analysis identified power spectral density and the first harmonic frequency as the most consistently influential features across both models and different activity classes, confirming the dominant role of frequency content in activity discrimination. For dynamic activities such as running and walking, frequency-domain features, including the second harmonic and its spectral location, were most discriminative, while stationary activities such as sitting and standing were primarily distinguished by the signal mean amplitude, reflecting the low-energy and stable nature of these movements.

Overall, well-engineered features combined with ensemble learning provide an accurate and interpretable approach to Human Activity Recognition using wearable sensors under the controlled, single-subject conditions evaluated in this study. From an implementation perspective, the results suggest that a practical wearable monitoring system could potentially integrate the proposed auxetic cTPU sensor with an embedded Random Forest classifier using a reduced set of SHAP-selected features, particularly power spectral density and harmonic frequency characteristics. These findings align with [25], where frequency features were shown to enhance the classification process and produce models with highly discriminative features, leading to higher prediction accuracy. Such a design may help reduce computational requirements while maintaining high classification accuracy and interpretability, representing a promising direction for future development toward real-time healthcare, rehabilitation, and human–robot interaction applications, pending validation on larger and more diverse datasets.

## 6. Limitations and Future Work

A key limitation of this study is the reliance on data from a single subject, which restricts the generalizability of the reported classification accuracies beyond the individual tested. The present evaluation was intentionally conducted as a controlled, single-subject proof-of-concept; consequently, the reported performance characterizes the discriminative capability of the proposed sensor–feature–classifier pipeline under these experimental conditions rather than its generalizability across users. The clear and physically interpretable feature separability revealed by the SHAP analysis offers encouraging preliminary evidence that extending this work is likely to succeed. The four activities tested are the most common movements in daily life and rehabilitation, and the repeated transitions in the recorded sequence give an early sign that the system can handle switching between activities.

Future work will focus on extending this framework to a larger, more diverse cohort using leave-one-subject-out cross-validation to assess performance across users, implementing the system on embedded hardware for real-time activity recognition, expanding the study to include additional activities, and evaluating the long-term performance and durability of the sensor under regular daily use and varying operating conditions. Furthermore, future work will focus on extended cyclic durability and long-term baseline-drift testing of the sensor to demonstrate long-term durability and reproducibility.

## Figures and Tables

**Table 1:** Three-dimensional printing parameter settings.

Parameter	Setting
Bed Temperature (°C)	50
Nozzle Temperature (°C)	260
Nozzle Diameter (mm)	0.4
Layer Height (mm)	0.2
First Layer Height (mm)	0.2
Infill Density (%)	100
Infill Pattern (%)	Monotonic
Print Speed (mm/s)	25
Cooling (%)	100

**Table 2:** Data collected for the Auxetic Sensors.

Parameter	Sample 1	Sample 2	Sample 3	Mean	STD	CV (%)
Gauge Factor (GF)	−0.35	−0.18	−0.19	−0.24	0.09	37.98
ΔR	−89.00	−35.00	−33.00	−52.33	31.77	–
$\Delta R/R_{0}$	−0.073	−0.035	−0.039	−0.049	0.021	–
Strain (ε) (mm/mm)	0.21	0.19	0.20	0.20	0.01	–
Sensitivity (ΔR/Δε)	423.81	184.21	165.00	257.67	144.20	–
Max Stress (σ) (MPa)	0.200	0.185	0.188	0.19	0.01	–
Young’s Modulus (*E*) (MPa)	0.95	0.97	0.94	0.96	0.02	–

**Table 3:** Performance comparison of Random Forest and XGBoost classifiers for Human Activity Recognition, based on cross-validation accuracy and training time.

Model	Accuracy (CV)	Train Time
Random Forest	100.00%	2.74 s
XGBoost	97.33%	0.27 s

**Table 4:** Effect of interpolation and normalization method on classification accuracy across memory lengths.

Interpolation	Normalization	Memory Length	Accuracy (%)
No	Relative mean	10 hidden points	91.4%
No	Linear	10 hidden points	86.0%
No	Z-score	10 hidden points	83.0%
No	Bipolar	10 hidden points	77.23%
No	No	10 hidden points	76.5%
No	Relative standard deviation	10 hidden points	76.0%
No	Binary	10 hidden points	73.4%
Yes	No	10 hidden points	73.0%
No	Relative standard deviation	50 hidden points	96.0%
No	Bipolar	50 hidden points	95.5%
No	Relative mean	50 hidden points	95.0%
No	Z-score	50 hidden points	94.4%
No	No	50 hidden points	94.0%
No	Linear	50 hidden points	90.3%
No	Binary	50 hidden points	86.2%
Yes	No	50 hidden points	73.0%
No	No	100 hidden points	94.87%
No	Relative standard deviation	100 hidden points	92.50%
No	Bipolar	100 hidden points	90.27%
No	Z-score	100 hidden points	90.0%
No	Linear	100 hidden points	74.8%
No	Relative mean	100 hidden points	76.2%
No	Binary	100 hidden points	74.6%
Yes	Relative mean	100 hidden points	74.6%
Yes	No	100 hidden points	67.0%
Yes	Relative standard deviation	100 hidden points	66.0%

## Data Availability

The data supporting the findings of this study are available from the corresponding author upon request.
